# Use of transgenic *Aedes aegypti* in Brazil: risk perception and assessment

**DOI:** 10.2471/BLT.16.173377

**Published:** 2016-08-31

**Authors:** Paulo Paes de Andrade, Francisco José Lima Aragão, Walter Colli, Odir Antônio Dellagostin, Flávio Finardi-Filho, Mario Hiroyuki Hirata, Amaro de Castro Lira-Neto, Marcia Almeida de Melo, Alexandre Lima Nepomuceno, Francisco Gorgônio da Nóbrega, Gutemberg Delfino de Sousa, Fernando Hercos Valicente, Maria Helena Bodanese Zanettini

**Affiliations:** a Departamento de Genética, Universidade Federal de Pernambuco, Avenida Moraes Rego s/s, 50670-901, Recife, Brazil.; b Embrapa, Brasília, Brazil.; c Universidade de São Paulo, São Paulo, Brazil.; d Universidade Federal de Pelotas, Pelotas, Brazil.; e Instituto Agronômico de Pernambuco – IPA, Recife, Brazil.; f Universidade Federal de Campina Grande, Patos, Brazil.; g Faculdade Anhanguera, Brasília, Brazil.; h Universidade Federal do Rio Grande do Sul, Porto Alegre, Brazil.

## Abstract

The OX513A strain of *Aedes aegypti*, which was developed by the British company Oxitec, expresses a self-limiting transgene that prevents larvae from developing to adulthood. In April 2014, the Brazilian National Technical Commission on Biosafety completed a risk assessment of OX513A and concluded that the strain did not present new biological risks to humans or the environment and could be released in Brazil. At that point, Brazil became the first country to approve the unconstrained release of a genetically modified mosquito. During the assessment, the commission produced a comprehensive list of – and systematically analysed – the perceived hazards. Such hazards included the potential survival to adulthood of immature stages carrying the transgene – should the transgene fail to be expressed or be turned off by exposure to sufficient environmental tetracycline. Other perceived hazards included the potential allergenicity and/or toxicity of the proteins expressed by the gene, the potential for gene flow or increased transmission of human pathogens and the occupation of vacant breeding sites by other vector species. The Zika epidemic both elevated the perceived importance of *Ae. aegypti* as a vector – among policy-makers and regulators as well as the general public – and increased concerns over the release of males of the OX513A strain. We have therefore reassessed the potential hazards. We found that release of the transgenic mosquitoes would still be both safe and of great potential value in the control of diseases spread by *Ae. aegypti,* such as chikungunya, dengue and Zika.

## Résumé

La souche OX513A d'*Aedes aegypti*, qui a été créée par la société britannique Oxitec, exprime un transgène autolimitant qui empêche les larves de se développer et de devenir adultes. En avril 2014, la Commission technique nationale de biosécurité du Brésil a procédé à une évaluation des risques liés à la souche OX513A et conclu qu'elle ne présentait pas de nouveaux risques biologiques pour les êtres humains ou l'environnement et pouvait être lâchée au Brésil. Le Brésil est donc devenu le premier pays à approuver le lâcher non contraint d'un moustique génétiquement modifié. Au cours de l'évaluation, la commission a établi une liste exhaustive des risques perçus, qu'elle a par ailleurs systématiquement analysés. Ces risques incluaient la survie potentielle à l'âge adulte des larves immatures porteuses du transgène – si le transgène ne s'exprime pas ou est désactivé par une exposition à la tétracycline suffisante dans l'environnement. Les autres risques perçus incluaient les potentielles propriétés allergisantes et/ou la toxicité des protéines exprimées par le gène, l'éventualité d'un flux de gènes ou d'une transmission accrue d'agents pathogènes pour l'homme et l'occupation de sites de reproduction vacants par d'autres espèces vectrices. L'épidémie d’infections à virus Zika a accentué l'importance accordée par les responsables politiques, les organismes de réglementation ainsi que le grand public à *Ae. aegypti* en tant que moustique vecteur, et a accru l'inquiétude relative au lâcher de mâles de la souche OX513A. Nous avons donc réévalué les risques potentiels. Nous estimons que le lâcher de moustiques transgéniques serait à la fois sans danger et extrêmement utile pour lutter contre les maladies transmises par *Ae. aegypti*, telles que le chikungunya, la dengue et le virus Zika.

## Resumen

La cepa OX513A de *Aedes aegypti*, que desarrolló la empresa británica Oxitec, expresa un transgén autolimitado que impide que las larvas se desarrollen hasta la edad adulta. En abril de 2014, la Comisión Nacional Técnica de Bioseguridad de Brasil realizó una evaluación de riesgos de OX513A y concluyó que la cepa no presentaba nuevos riesgos biológicos para los humanos o el medioambiente y que podría liberarse en Brasil. En ese momento, Brasil se convirtió en el primer país en aprobar la liberación ilimitada de un mosquito modificado genéticamente. A lo largo de la evaluación, la comisión redactó una lista completa, y analizada sistemáticamente, de las posibles contingencias. Entre dichos peligros se encontraba la posible supervivencia hasta la edad adulta de etapas inmaduras que portan el transgén, en caso de que éste no consiga expresarse o se inutilice debido a la exposición a la suficiente tetraciclina medioambiental. Otras posibles contingencias eran la alergia y/o toxicidad de las proteínas expresadas por el gen, la posibilidad de un flujo genético o el aumento de la transmisión de patógenos humanos y la ocupación de lugares de cría desocupados por parte de otras especies vectores. La epidemia por el virus de Zika aumentó la importancia de *Ae. aegypti *como vector, entre los responsables y reguladores políticos, así como entre el público general, y aumentó las preocupaciones acerca de la liberación de machos de la cepa OX513A. Por lo tanto, se han vuelto a evaluar los posibles riesgos. Se ha descubierto que la liberación de mosquitos transgénicos sería segura y tendría un gran valor potencial en el control de la propagación de enfermedades por *Ae. aegypti*, como la fiebre chikungunya, el dengue y la enfermedad por el virus de Zika.

## ملخص

تمثل سلالة OX513A من *بعوضة الحمى الصفراء *، والتي تم تطويرها عن طريق الشركة البريطانية Oxitec، أحد الجينات المعدّلة وراثيًا ذاتية الحد والتي تمنع اليرقات من التطور إلى فترة البلوغ. وفي شهر أبريل/نيسان من عام 2014، قامت اللجنة الفنية الوطنية البرازيلية للسلامة البيولوجية بإتمام إجراء تقييم للمخاطر يتعلق بسلالة OX513A، وخلصت اللجنة إلى أن السلالة لا تمثل أي مخاطر بيولوجية جديدة للإنسان أو البيئة، ويمكن أن يتم إطلاقها في البرازيل. وأصبحت البرازيل في تلك اللحظة أول دولة توافق على إطلاق البعوض المعدّل وراثيًا من دون قيود. قامت اللجنة في أثناء التقييم بإنشاء قائمة شاملة تضم المخاطر الملحوظة والشروع في تحليلها بشكل منهجي. واشتملت بعض هذه المخاطر على احتمالية نجاة البعوض في مراحل عدم النضج التي تحمل الجين المعدّل وراثيًا إلى فترة البلوغ – في حال فشل الجين المعدّل وراثيًا في التعبير عن نفسه أو توقف عن العمل نتيجة لتعرضه لكمية كافية من التتراسيكلين الموجود في البيئة. واشتملت بعض المخاطر الملحوظة الأخرى على احتمالية وجود عناصر مسببة للحساسية و/أو السمية في البروتينات التي تعبر عنها الجينات، واحتمالية تدفق الجينات أو الانتقال الزائد لمسببات الأمراض البشرية، واحتلال مواقع التكاثر الشاغرة بواسطة أنواع ناقلات الأمراض الأخرى. لقد ساهم وباء زيكا في رفع درجة الأهمية الملحوظة *لبعوضة الحمى الصفراء * كإحدى ناقلات الأمراض - بين صنّاع القرار وواضعي اللوائح التنظيمية فضلاً عن عامة الناس - وزيادة المخاوف بشأن إطلاق الذكور من سلالة OX513A. ولذلك قمنا بإعادة تقييم المخاطر المحتملة. لقد وجدنا أن إطلاق البعوض المعدّل وراثيًا لا يزال وسيلة آمنة ويمثل قيمة محتملة كبيرة في مكافحة الأمراض المنتشرة عن طريق *بعوضة الحمى الصفراء *، مثل داء شيكونغونيا وحمى الضنك وفيروس زيكا.

## 摘要

OX513A 类*埃及伊蚊*由英国 Oxitec 公司培育而成，该蚊携带自我限制的转基因，能够阻止幼虫生长为成虫。 2014 年 4 月，巴西国家生物安全技术委员会 (Brazilian National Technical Commission on Biosafety) 完成了一项对 OX513A 的风险评估，评估结果表明该品种不会对人类或环境带来新的生物风险，并且可以在巴西释放。 当时，巴西成为了第一个批准无限制释放转基因蚊子的国家。 评估期间，该委员会系统制定了一份已知危害的详细列表，并对其进行了系统分析。 此类危害包括在尚未成熟阶段携带转基因的成虫可能存活下来——转基因未成功携带或由于暴露于充足的四环素环境而被抑制。 其他已知危害包括基因中携带的蛋白质具有致敏性和/或毒性、潜在的基因流动或人类病原体传播加剧，以及空置繁殖场所被其它病媒物种占用。 寨卡病毒不仅提高了政策制定者、监管部门以及公众对*埃及伊蚊*的重要性的认识，同时也增加了对释放 OX513A 类雄性蚊子的担忧。 因此，我们重新评估了潜在危害。 我们发现，释放转基因蚊子目前仍然安全，并且在控制通过*埃及伊蚊*传播的疾病（例如基孔肯雅热、登革热和寨卡等病毒性疾病）方面具有很大的潜在价值。

## Резюме

Вид комаров Aedes aegypti под названием OX513A, разработанный британской компанией Oxitec, экспрессирует ограничивающий популяцию своего носителя трансген, который препятствует развитию личинки во взрослую особь. В апреле 2014 года Бразильская национальная техническая комиссия по биобезопасности завершила оценку рисков OX513A и пришла к выводу, что этот вид не представляет новых биологических угроз для человека или окружающей среды и может быть выпущен в Бразилии. Тогда Бразилия стала первой страной, в которой был разрешен неограниченный выпуск генетически измененных комаров. В ходе оценки комиссия опубликовала исчерпывающий перечень предполагаемых рисков и провела их систематический анализ. Такие риски включали потенциальную способность комаров неполовозрелой стадии, несущих трансген, дожить до репродуктивного возраста в случае неудавшейся экспрессии трансгена или его подавления, вызванного воздействием достаточного количества тетрациклина на комаров во внешней среде. Среди других предполагаемых рисков — потенциальная аллергенность или токсичность белков, образующихся в результате экспрессии гена, возможность дрейфа генов или повышенного распространения болезнетворных организмов для человека и заселение свободных мест размножения другими видами переносчиков. На фоне эпидемии вируса Зика лица, ответственные за разработку стратегических решений, регулирующие органы, а также общественность увеличили внимание к роли Ae. Aegypti в качестве переносчиков и выразили большую обеспокоенность по поводу выпуска мужских особей вида OX513A. По этой причине авторы данной статьи провели повторную оценку потенциальных рисков. Была подтверждена безопасность и высокое потенциальное значение выпуска генетически измененных комаров для борьбы с распространяемыми Ae. aegypti заболеваниями, такими как чикунгунья, денге и болезнь, вызываемая вирусом Зика.

## Introduction

In April 2014, Brazil’s National Technical Commission on Biosafety –the agency officially responsible for the assessment of the risks posed by genetically modified organisms in Brazil – assessed the potential risks of the release in Brazil of the transgenic OX513A strain of *Aedes aegypti* and concluded that such a release would be safe.^1^ At that point, Brazil became the first country to approve the unconstrained release of a genetically modified mosquito. Two years later, however, the Zika epidemic had added to the general public’s concerns over the release of mosquitoes and we therefore decided to re-investigate the perceived risks and update the commission’s risk assessment.

## Control of arbovirus vectors

Several arboviruses – for example chikungunya virus, dengue viruses and, more recently, Zika virus – cause much human suffering in Brazil.^2^^,^^3^ In 2015, for example, there were more than 1.5 million suspected cases of dengue fever in the country.^2^ While attempts to create effective or cost–effective vaccines continue, control of the mosquito vectors remains of the utmost importance.^4^ Brazil has had some success in controlling mosquitoes, even early in the 20th century when no effective insecticides were available.^5^ Mosquito control, by residents, health workers and other municipal workers supported by a heterogeneous and broad set of collaborators, remains the focus of the National Dengue Control Programme.^6^ However, the failure of this programme to reduce vector populations to levels that could interrupt dengue transmission^7^ has spurred Brazilian interest in dengue vaccines^8^ and novel approaches to vector control such as the sterile insect technique.^9^^–^^13^ The International Atomic Energy Agency describes the sterile insect technique as “a type of birth control in which wild female insects of the pest population do not reproduce when they are inseminated by released, radiation-sterilized males”.^14^ Although sequential releases of large numbers of the sterilized males should lead to a reduction in the size of the pest population, the irradiation used can reduce the released insects’ competitiveness – and this appears to be a particular problem when the insects involved are mosquitoes.^15^ Over the last decade, as an alternative to the sterile male technique, the genetic modification of mosquitoes, with the production of large numbers of males and females that carry a self-limiting transgene, has been investigated.^16^ Once released in the environment, the male insects carrying the transgene – which have to be produced in the presence of a selective agent that blocks the transgene’s expression – should, potentially, compete on equal terms with the wild males.^17^^–^^20^

**Fig. 1:**
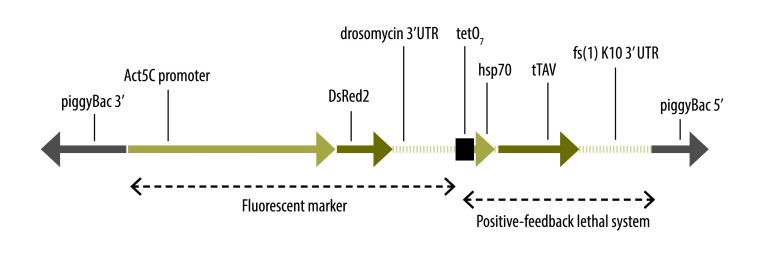
Structure and function of the OX513 insert, a transgenic construction inserted into the *Aedes aegypti* genome

The OX513A strain of *Ae. aegypti*, which was developed by the British company Oxitec, expresses a self-limiting dominant transgene that is able to kill, at larval stage, the mosquitoes in which it is expressed (Fig. 1).^21^ In the presence of tetracycline, the transgene is either not expressed or is only expressed at a very low and non-lethal level. Once released in the environment, most males carrying the transgene die after about two days.^21^

tTAV: tetracycline-repressible transcriptional activator; UTR: untranslated region.

Notes: The insert is a non-autonomous transposon derived from a piggyBac transposon. Transformants may be traced via the fluorescent protein expressed by the DsRed2 gene, which expression is driven by the Act5C promoter. The tetracycline-repressible transcriptional activator is under control of its own binding site, tetO_7_ – a minimal *Drosophila* hsp70 promoter. The increase in the intracellular concentration of this activator disrupts cell metabolism and leads to cell death. Although the insert’s 5’ and 3’ ends allow the insert’s insertion in double-stranded deoxyribonucleic acid in the presence of a specific transposase, the insert cannot be further mobilized because the necessary enzyme is not expressed by the transgenic mosquito. Drosomycin 3’ UTR and fs(1)K103’ UTR are spacers.^21^

## Risk perceptions and assessments

In almost all countries, any living modified organism derived from modern biotechnology is very strictly regulated and subjected to a long and detailed risk assessment.^22^ In Brazil, which is a Party to the Cartagena Protocol on Biosafety to the Convention on Biological Diversity, such risk assessment is the sole responsibility of National Technical Commission on Biosafety.^23^ The commission consists of 27 members and an equal number of surrogates – chosen by various federal government agencies and civil organizations – including highly qualified scientists from across the country. As stipulated in the Cartagena Protocol, any risk assessment of a genetically modified product has to be supported with so-called hard data and preferably with hard data collected in the country that intends to use the product. In 2011, the commission was asked to conduct a risk assessment of the biosafety of the OX513A strain of *Ae. aegypti* as a precursor to the experimental, small-scale release of adult males of the strain in Brazil’s Bahia state –first in Juazeiro and then in Jacobina– that would take place from 2012 to 2014. The commission used data collected in these releases and in field experiments conducted in the Cayman Islands and Malaysia, along with some relevant laboratory results, to produce a comprehensive risk assessment.^1^ In April 2014, on the basis of this assessment, the commission approved the OX513A strain for unconstrained release throughout Brazil, primarily as a method of dengue control.

Risk assessments by the commission, like those by most agencies tasked with assessing the risks posed by the release of a genetically modified organism, consist of four main steps, which comply with the Cartagena Protocol: (i) problem formulation; (ii) risk characterization; (iii) risk classification; and (iv) an overall safety evaluation.^24^^,^^25^ In problem formulation, specialists outline the release scenario and list all the associated hazards – as perceived by themselves, by other specialists and by the general public. Each perceived hazard is then assessed in the context of the planned release, using any relevant data that are available and considering each plausible route by which the hazard may cause harm. In this risk characterization, any perceived hazards that cannot plausibly lead to harm are excluded. In the subsequent risk classification, the risk of each of the remaining perceived hazards occurring is estimated. Finally, the assessors estimate the overall risk posed by the planned release and consider whether the organism should be considered safe for the environment.

Although international guidelines on the assessment of food safety^26^ do not apply directly to mosquitoes, they can still be useful in assessing the allergenicity and toxicity of novel proteins created as the result of the genetic modification of such insects.

### 2014 assessment

As part of its risk assessments of the OX513A strain in 2014, the Technical Commission listed the potential hazards. Although some of the listed hazards were perceived by specialists, most had been voiced by the general public. Among the main perceived hazards were the unexpected survival of at least some of the mosquitoes that carried the transgene and/or their progeny as the result of partial penetrance and/or the presence of tetracycline in the environment, allergenicity and/or toxicity of two new proteins expressed, possibly in the mosquitoes’ saliva, as a result of the genetic modification, the vertical and horizontal flow of the transgene and its consequences, the participation of transgenic mosquitoes in the transmission of dengue viruses and the occupation, by other vector species, of breeding sites made vacant by the intervention.^1^

Only one of the listed perceived hazards – that is, survival in the presence of tetracycline – was considered plausible, and even that hazard was deemed unlikely since concentrations of tetracycline found in freshwater are usually more than two orders of magnitude lower than that needed to block the transgene’s expression in laboratory-bred insects.^1^ In addition, only 50% of the progeny of any female mosquitoes carrying the transgene into adulthood would carry the lethal gene – and they all die at the larval stage if not also protected by exceptionally high concentrations of tetracycline.

Although the occupation of breeding sites cleared of *Ae. aegypti* by other vector species – for example *Ae. albopictus* – was deemed a negligible risk by the entomologists involved in the risk assessment, it remained an area of concern among a few of the commission’s non-specialists.

The commission approved the nationwide release of adult males of the OX513A strain on the condition that Oxitec investigated tetracycline concentrations in potential breeding sites by means of periodic literature reviews and also investigated the occupation of cleared breeding sites by *Ae. albopictus*.

### 2016 reassessment

By 2016, the recent Zika outbreak had led to additional concerns about the release of any *Ae. aegypti* in Brazil, although few of these concerns had been voiced by universities, research centres or official risk assessment agencies. Such new concerns had arisen even though, by 2016, the usefulness of genetic modification in the control of mosquitoes had been demonstrated in Brazil and elsewhere^17^^,^^19^^,^^27^ and there appeared to be ever-growing acceptance of such an approach to vector control among the general public and public health managers. Among the new concerns were the potential participation of released mosquitoes or their progeny in the transmission of Zika virus, and the perceived possibility of horizontal gene transfer from the transgenic mosquitoes to the Zika virus – potentially making the virus more harmful to humans. Although to us neither of these newly perceived adverse effects of the release of transgenic mosquitoes in Brazil appeared plausible, in April 2016 we reassessed the general safety of the unconstrained release of males of the OX513A strain of *Ae. aegypti* in Brazil.

**Fig. 2:**
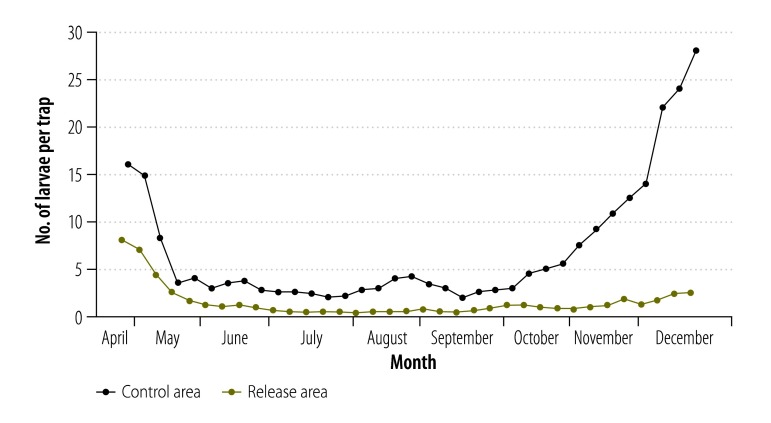
Numbers of mosquito larvae in traps set up in a control area where males of the transgenic OX513A strain of *Aedes aegypti* were released, Piracicaba county, Brazil, 2015

Notes: The transgenic male mosquitoes were released throughout 2015. Data shown here were previously presented.^28^Figure republished with permission from Oxitec, Abingdon, United Kingdom of Great Britain and Northern Ireland.

The plans for the commercial releases of males of the OX513A strain throughout Brazil have not been changed since the 2014 risk assessment. It is expected that the releases will take place mainly in cities or other densely populated areas, in association with other control measures and under continuous surveillance by the relevant municipal health authorities and Oxitec. At the time of writing, a large-scale but still pre-commercial release is in progress in Piracicaba county in São Paulo state. The data already collected during this release (Fig. 2) confirm the effectiveness of such releases, support the encouraging results previously obtained in the Cayman Islands, Malaysia and the Brazilian town of Juazeiro,^19^^,^^27^ and the commission’s earlier conclusion that such releases are safe.

As male mosquitoes do not feed on blood, the intention is to release only male *Ae. aegypti.* However, there is a small margin of error during the separation of males and females before field release and therefore up to 0.2% of the released insects may be females.^20^Although the bites of the released females are clearly perceived as a risk by the general public, they are relatively rare and most of the released females will be dead within two days, too soon for them to become infectious even if they have taken a blood meal that contains chikungunya virus, dengue virus or Zika virus.^29^ Even if, as may occasionally happen, a female carrying the transgene survives long enough to become infectious, there is no evidence that it will be any worse as a vector than a wild *Ae. aegypti*.^30^

As genomic analyses have revealed many examples of gene transfer between distinct taxa,^31^ horizontal gene transfer is always a controversial issue in discussions about the unanticipated risks of releasing a genetically modified organism. However, such gene transfers appear to be very infrequent. There is evidence of horizontal gene transfers from invertebrates to mammals but all such transfers that have been detected appear to be transposon-dependent and very infrequent.^32^^–^^34^ It does not seem possible that the piggyBac transposon used in the construction of the OX513A strain of *Ae. aegypti* could mediate the transfer of genetic information from the mosquito to other genomes. This transposon does not codify transposase and, since transposase is not codified in the mosquito’s genome, the insert cannot be remobilized.

Even if a transposase were present, there seems to be no risk of genetic transfer between the OX513A strain’s transgene and the Zika virus because the viral genome is made of single-stranded ribonucleic acid, which is not a substrate for transposases. When, in an effort to understand the Zika outbreak in Brazil, various isolates of the Zika virus were sequenced, no evidence was found for transposon insertion or any other form of horizontal gene transfer.^35^

## Conclusion

After circulating around the world, the Zika virus is rapidly spreading through the Americas.^36^ The detection of an association between microcephaly in a baby and maternal infection with Zika virus^37^^–^^41^ has greatly increased the perceived importance of the primary vector of Zika virus – that is, *Ae. aegypti* – among both the general public and policy-makers.^42^ The results of our reassessment of the safety of releasing males of the genetically modified OX513A strain of *Ae. aegypti,* as well as the results of a related assessment by the United States Food and Drug Administration,^43^ indicate that releases of such mosquitoes still offer a safe and potentially effective way of reducing wild populations of the vector. The risk-assessment components adopted by both the commission and the United States Food and Drug Administration^43^ are those recommended by the World Health Organization.^44^ In the ongoing battle against the Zika outbreak, the Brazilian health managers’ choice of control methods has to be based on careful risk assessments and not on risk perceptions. Public perceptions – especially with the advent of fast and global communications via the Internet – are often inaccurate and the general public is often misled by conspiracy theories and catastrophism.^45^^–^^47^ In the field of mosquito control by genetic modification, Brazil is a world leader. Brazil has already evaluated the OX513A strain of *Ae. aegypti* and approved the strain’s commercial use. Once the strain has been registered at Brazil’s National Health Surveillance Agency, it will probably be commercially released throughout the country.
